# Characterization of Temperature and Strain Changes in Lithium-Ion Batteries Based on a Hinged Differential Lever Sensitization Fiber Bragg Grating Strain–Temperature Simultaneous-Measurement Sensor

**DOI:** 10.3390/s24020412

**Published:** 2024-01-10

**Authors:** Meng Li, Weigen Chen, Zhiwei Shen, Ziyi Wang, Zifeng Ming, Changding Wang, Haoyuan Tian, Tianyi Sang, Ruimin Song

**Affiliations:** State Key Laboratory of Power Transmission Equipment Technology, School of Electrical Engineering, Chongqing University, Chongqing 400044, China; limeng123@cqu.edu.cn (M.L.); 20231101051@stu.cqu.edu.cn (Z.S.); wzycsust@163.com (Z.W.); 202211131152t@stu.cqu.edu.cn (Z.M.); wangchd@cqu.edu.cn (C.W.); hytian@cqu.edu.cn (H.T.); richardsty@163.com (T.S.); ruiminsong@cqu.edu.cn (R.S.)

**Keywords:** Li-ion battery (LIB), fiber Bragg grating (FBG) sensors, strain, temperature, overcharge of the battery, enhanced-sensitivity sensors

## Abstract

Li-ion batteries are expected to become the mainstream devices for green energy storage or power supply in the future due to their advantages of high energy and power density and long cycle life. Monitoring the temperature and strain change characteristics of Li-ion batteries during operation is conducive to judging their safety performance. The hinged differential lever sensitization structure was used for strain sensitization in the design of an FBG sensor, which also allowed the simultaneous measurement of strain and temperature. The temperature and strain variation characteristics on the surface of a Li-ion soft-packed battery were measured using the des.igned sensor. This report found that the charging and discharging processes of Li-ion batteries are both exothermic processes, and exothermic heat release is greater when discharging than when charging. The strain on the surface of Li-ion batteries depends on electrochemical changes and thermal expansion effects during the charge and discharge processes. The charging process showed an increasing strain, and the discharging process showed a decreasing strain. Thermal expansion was found to be the primary cause of strain at high rates.

## 1. Introduction

With the development and progress of technology, the human demand for energy is gradually increasing, and the search for safe and reliable new energy sources has become an urgent problem [1,2]. Storage and on-demand utilization of electrical energy are critical, and Li-ion batteries are excellent energy storage carriers [3]. Li-ion batteries have the unique advantages of high energy and power density, high energy conversion efficiency, long cycle life, low production cost, and low environmental pollution [4,5]. Li-ion batteries have a wide range of application prospects and are expected to become the mainstream equipment for green energy storage or power supply in the future [6,7].

However, during the charging and discharging processes of Li-ion batteries, safety issues such as battery bulging and expansion, failure, and thermal runaway occur from time to time, seriously affecting the development of the industry and personal safety [8,9,10]. It is crucial to realize the safety testing of Li-ion batteries and determine the strain, stress, and temperature changes during Li-ion battery operation. Stress or surface strain changes in Li-ion batteries include volume expansion associated with the two electrode interludes, phase change at the cathode, SEI formation at the anode, and thermal expansion/contraction due to internal resistance heating and electrochemical reactions [11,12,13]. The expansion of the cell is not a square thickness expansion, but a bulging expansion. The main manifestation of this is the lateral parallel expansion of the cell surface. The determination of the degree of expansion based on the measurement of strain coefficients is one of the most important methods in the measurement of battery condition coefficients. The heat generated in Li-ion batteries is mainly composed of four elements: reaction heat, Joule heat, polarization internal resistance heat, and side-reaction heat. When the exothermic rate of the Li-ion battery exceeds the rate of heat dissipation, the internal temperature of the battery will gradually increase, the exothermic reaction will intensify, and the accumulation of heat to the critical point will cause thermal runaway [14,15,16]. Changes in temperature and strain parameters of Li-ion batteries can reflect changes in battery capacity, state of charge, and other parameters [17,18,19]. Therefore, the safety performance of the Li-ion battery can be judged by detecting the changes in temperature and strain in the battery during running. Some sensors are used to detect the temperature and strain parameters of Li-ion batteries [20]. Zhu et al. designed an encapsulated thin-film strain sensor with electrolyte corrosion resistance to demonstrate for the first time the evolution of internal strain during the charging and discharging of 18,650 Li-ion batteries [21]. Zhang et al. reported a flexible sensor array for Li-ion batteries with a fast reversible temperature switch that can be integrated into the battery to warn of thermal runaway [22]. The optical fiber sensor has the characteristics of anti-electromagnetic interference, electrical insulation, corrosion resistance, etc., and is suitable for the quantitative detection of characteristic parameters of Li-ion batteries [23]. The sensor based on fiber Bragg grating (FBG) adopts wavelength modulation, is easy to use, and is not affected by intensity noise [24,25,26]. Several methods have been proposed for cell temperature and strain detection based on the fiber optic sensing technology [27,28]. Laura et al. embedded an FBG into a button battery containing liquid or solid electrolytes and monitored the light signal during the battery cycle, thus realizing the detection of the internal pressure of the button battery [29]. Peng et al. proposed a novel high-precision strain-monitoring method based on FBG sensors with higher sensitivity than bare fiber sensors and explored the relationship between strain and SOC/DOD using the designed sensors [30]. Ee et al. used an FBG to simultaneously measure temperature and strain in Li-ion batteries, improved the detection accuracy using the PHRI method, and estimated the SOC based on a DNN [31]. Many of the proposed methods are important for the detection of temperature and strain in Li-ion batteries. Table 1 summarizes the many various FBG sensing methods used for Li-ion soft-packed battery applications. The proposed methods demonstrate the feasibility of optical fiber materials for temperature and strain detection in Li-ion batteries. However, there are some shortcomings at present. For example, fiber optic sensors are susceptible to temperature interference and have high manufacturing costs, and their sensitivity and other parameters need to be corrected in certain applications. Simultaneously, the designed FBG sensors are not sensitive enough, the detection of their temperature and strain is complex, and the analysis of the battery data is inadequate. Therefore, it is extremely meaningful to improve FBG sensors and enhance the comprehensiveness of the detection data in research on Li-ion battery parameter detection.

In this study, we designed a hinged differential multi-stage lever amplification mechanical transducer sensitization structure for battery surface strain based on the FBG sensing technology. Combined with the FBG tandem multiplexing technology, the simultaneous strain and temperature measurement on the surface of Li-ion soft-packed batteries was realized. Li-ion batteries’ temperature and strain were detected under regular and high-rate charging and discharging. The strain and temperature changes of Li-ion soft-packed batteries under various operating conditions were investigated.

## 2. Materials and Methods

### 2.1. FBG Sensing and Temperature/Strain Measurement Principles

An FBG is an optical passive device. A broadband light source is used as a laser source, and light is incident along the fiber grating axis through the fiber core. In the grating region, broadband light is mode-coupled when resonance is satisfied. The grating structure allows light that meets the Bragg wavelength conditions to be reflected, and the remaining wavelengths are transmitted and continue to propagate along the fiber core. Changes in external parameters will affect the effective refractive index $n_{eff}$ and the grating period Λ of the core of the fiber grating structure. The corresponding fiber Bragg wavelength $\lambda_{B}$ will also change, and the expression for the wavelength at the center of the reflection spectrum of the FBG is:

(1) $$\lambda_{B}=2n_{eff}\Lambda ,$$

When the FBG is subjected to external strain changes, the elasto-optical coefficient $P_{e}$ of the fiber material changes, which in turn causes the fiber Bragg wavelength to drift, i.e.,

(2) $$\Delta \lambda_{B\varepsilon }=(1-P_{e})\varepsilon \cdot \lambda_{B},$$

In the formula, $\Delta \lambda_{B\varepsilon }$ is the drift of the center wavelength of the reflected light due to the strain, and *ε* is the change in the external strain. From Equation (2), it can be seen that the center wavelength drift of the reflected light varies linearly with the external strain, and the corresponding external strain can be obtained by solving the center wavelength drift to realize strain demodulation.

When the FBG is subjected to external temperature changes, the thermo-optic effect will cause changes in the refractive index of the fiber core of the FBG. The thermal expansion effect will also cause changes in the grating region of the grating period; the change in the wavelength of the center of the reflected light caused by the temperature change can be expressed as:

(3) $$\Delta \lambda_{BT}=(\alpha_{s}+\xi_{s})T\cdot \lambda_{B},$$

where $\Delta \lambda_{BT}$ is the amount of drift in the central wavelength of the reflected light due to strain; $\alpha_{s}$ is the thermal expansion coefficient of the fiber; $\xi_{s}$ is the thermo-optic coefficient of the fiber; and *T* is the external temperature. Since the coefficient of thermal expansion of the FBG material changes little, the effect of temperature on the coefficient of thermal expansion can be ignored; so, the external temperature *T* can be expressed as:

(4) $$T=\frac{1}{\lambda_{B}\xi_{s}}\cdot \Delta \lambda_{BT},$$

When the FBG is affected by external temperature and strain, various factors act on the fiber grating simultaneously, showing a superposition effect. Therefore, the change in reflected-light-center wavelength can be expressed as:

(5) $$\Delta \lambda_{B}=(1-P_{e})\lambda_{B}\cdot \varepsilon +\xi_{s}\lambda_{B}\cdot T,$$

Considering

(6) $$\left[\begin{array}{l} \Delta \lambda_{B1}\\ \Delta \lambda_{B2} \end{array}\right]=\left[\begin{array}{cc} (1-P_{e})\lambda_{B1} & \xi_{s}\lambda_{B1}\\ (1-P_{e})\lambda_{B2} & \xi_{s}\lambda_{B2} \end{array}\right]\cdot \left[\begin{array}{l} \varepsilon \\ T \end{array}\right]$$

and assuming $\left[\begin{array}{cc} (1-P_{e})\lambda_{B1} & \xi_{s}\lambda_{B1}\\ (1-P_{e})\lambda_{B2} & \xi_{s}\lambda_{B2} \end{array}\right]=K$, we have:

(7) $$\left[\begin{array}{l} \varepsilon \\ T \end{array}\right]=K^{-1}\cdot \left[\begin{array}{l} \Delta \lambda_{B1}\\ \Delta \lambda_{B2} \end{array}\right],$$

When the FBG material and the center wavelength are determined, $K^{-1}$ is a fixed value. Therefore, two different center wavelength gratings connected in series on an optical fiber can be used to measure the temperature and strain at the same location. In this case, the center wavelength of each grating changes linearly under the influence of external strain and temperature. The center wavelength shift of the two gratings can be used to achieve the simultaneous measurement of temperature and strain.

In practical engineering applications, temperature variations due to environmental factors are unavoidable. To facilitate the calibration of the sensor, one of the FBGs is only affected by the temperature. Get the temperature changes at the measurement point, i.e.,:

(8) $$T=\frac{1}{\xi_{s}\lambda_{B1}}\cdot \Delta \lambda_{B1},$$

Since the effect of both temperature and strain on the center wavelength shift is linear, the strain change at the measurement point can be obtained from the center wavelength shift on the other FBG, i.e.,

(9) $$\varepsilon =\frac{\Delta \lambda_{B2}}{(1-P_{e})\lambda_{B2}}-\frac{1}{(1-P_{e})\lambda_{B1}}\cdot \Delta \lambda_{B1}$$

### 2.2. Structure Design of the Temperature and Strain Sensor

#### 2.2.1. Design of a Hinged Differential Lever Amplification Sensitization Structure

**Figure 1:**
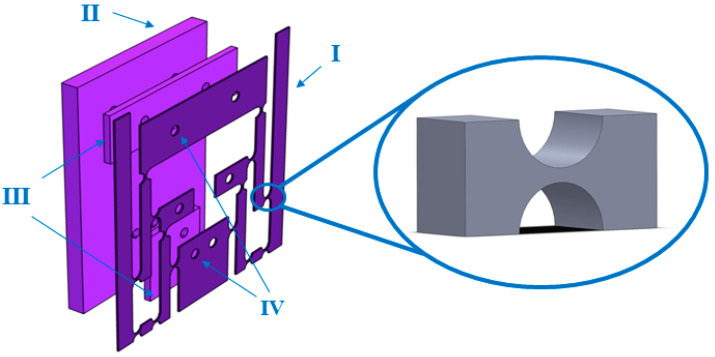
Hinged differential lever amplification sensitization structure and enlarged schematic diagram of the inter-rod hinge structure.

FBG sensors without a transducer structure have limited sensitivity to external strains, which often makes it difficult to meet practical measurement requirements. Therefore, the design of an externally amplified strain-sensitizing structure was considered. The designed hinged sensitization structure based on the differential lever amplification principle is shown in Figure 1, where I is the main element of the hinged differential lever amplification sensitization structure; II is a fixed plate used to maintain the stability of the overall structure; III is a dividing plate between the two structures; IV indicates customized threaded holes connecting each structure. When the external strain is transferred between adjacent rigid rods, a certain amount of material is removed by laser cutting from the rectangular or circular cross section connecting the two rigid rods, so that a weaker structure is formed between the rods. This ensures that the lever structure has a large flexibility and angle of rotation. It can also constrain the drift of the center of rotation at each rigid rod connection during deformation, therefore forming a flexible hinge structure. Hinged structures can be single-axis, double-axis, and multi-axis. Single-axis flexible hinges include straight circular, oval, hyperbolic, parabolic, and angular circular types. In the designed sensitized structure, we used a single-axis straight circular, flexible hinge.

**Figure 2:**
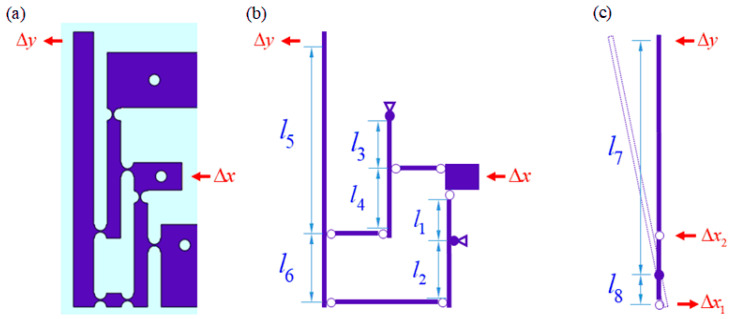
Schematic diagram of the main body of the hinged differential lever amplification sensitization structure. (**a**) Half of the sensitization body structure; (**b**) simplified diagram of the structure; (**c**) analytical diagram of the secondary differential lever.

The main part of the sensitized structure (spring steel) is symmetrical; so, only one side was mechanically analyzed, as shown in Figure 2a. A simplified schematic of the sensitizing structure is shown in Figure 2b. It consists of two first-stage levers and one second-stage lever. The two first-stage levers deform under the action of the displacement Δx. Based on the leverage effect, two deformations acting in opposite directions, $\Delta x_{1}$ and $\Delta x_{2}$, are produced on the stressed end of the second-stage lever, as shown in Figure 2c, with magnitudes

(10) $$\Delta x_{1}=\frac{l_{2}}{l_{1}}\Delta x,\Delta x_{2}=\frac{l_{3}+l_{4}}{l_{3}}\Delta x,$$

$\Delta x_{1}$ and $\Delta x_{2}$ have a differential effect on the second-stage lever structure, which shifts the center of rotation of the second-stage lever downward, thereby producing amplification. In practice, the shape variable Δx at the input is much smaller than the length of each lever. The irregular circular displacements of the force points generated by the unfixed end of the second-stage lever can be considered horizontal movements. The position of the new center of rotation can be determined based on the magnitude of the differential displacement:

(11) $$l_{7}=l_{5}+\frac{l_{1}l_{3}l_{6}+l_{1}l_{4}l_{6}}{l_{2}l_{3}+l_{1}l_{3}+l_{1}l_{4}},l_{8}=\frac{l_{2}l_{3}l_{6}}{l_{2}l_{3}+l_{1}l_{3}+l_{1}l_{4}},$$

The output deformation can be obtained as:

(12) $$\Delta y=\frac{\left(l_{5}+l_{6})(l_{2}l_{3}+l_{1}l_{3}+l_{1}l_{4}\right)}{l_{1}l_{3}l_{6}}\Delta x,$$

Thus, the total sensitization factor $K_{T}$ of this hinged differential amplifying lever structure is:

(13) $$K_{T}=\frac{\Delta y}{\Delta x}=\frac{\left(l_{5}+l_{6})(l_{2}l_{3}+l_{1}l_{3}+l_{1}l_{4}\right)}{l_{1}l_{3}l_{6}},$$

Displacement transfer will be affected if the hinge and multi-stage lever structure in the sensitized structure are too thin. Also, it is easy to produce mechanical damage and affect the elastic recovery when strain occurs. However, an excessive thickness of the sensitized structure may also affect the surface expansion of Li-ion soft-packed batteries during surface strain measurement. A balance was found when the thickness of the overall hinge and lever system in the main structure was 0.8 mm, i.e., neither too thick nor too thin.

For the entire sensitization structure, the higher the displacement amplification, the higher the force required. To ensure that the moment of deformation of the sensor input is not so large as to affect the actual effectiveness of the sensor, it is sufficient to ensure that the magnification of each lever stage is only within its applicable range. The parameters of the lever structure at each level were selected as $l_{1}=l_{2}=16\;\mathrm{mm}$, $l_{3}=l_{4}=16\;\mathrm{mm}$, $l_{5}=2.3l_{6}=36.8\;\mathrm{mm}$. The width of the designed lever structure was 4.2 mm. According to Equation (13), the sensitization factor was 9.9.

**Figure 3:**
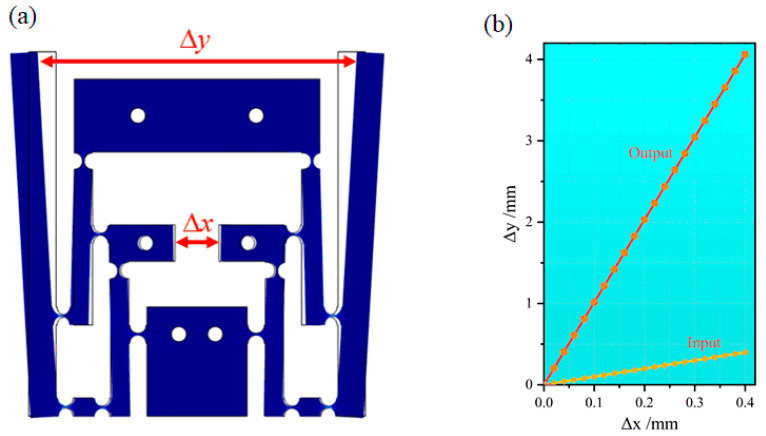
Schematic diagram of (**a**) deformation state and (**b**) magnification obtained in the strain-sensitized solid mechanics finite element simulation.

The corresponding hinge and lever models were established, and solid mechanics FEM simulation was carried out to obtain the deformation state and magnification of the structure when subjected to force, as shown in Figure 3. A schematic of the deformation state simulation of this sensitized structure is shown in Figure 3a. It can be clearly seen that the sensitized structure could indeed realize the amplification of displacement and strain, and its change state was consistent with the theoretical analysis. According to the parametric scanning FEM simulation analysis of the output end against the input end displacement (0–0.40 mm, interval 0.02 mm), the input and output curves converged, as shown in Figure 3b. It was calculated that the sensitized structure could achieve a 10-time-larger amplification effect. The slight difference between the FEM simulation results and those from the theoretical analysis of the amplification was due to the arc approximation that turned the curve shift into a linear shift in the theoretical analysis. The structure appeared theoretically effective in increasing the deformation of the measured structure, which in turn increased the sensitivity of the designed strain sensor.

#### 2.2.2. FBG Temperature–Strain Fusion Detection Structure Design

**Figure 4:**
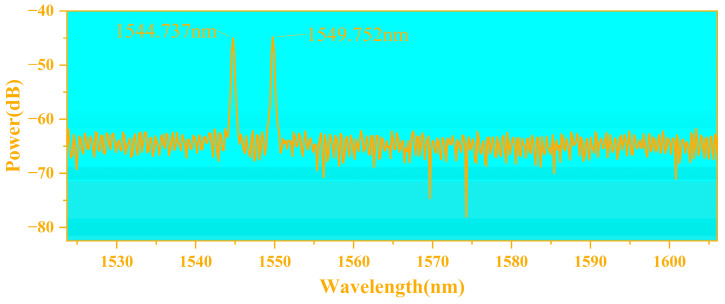
Reflectance spectra of two FBGs for temperature and strain measurements.

**Figure 5:**
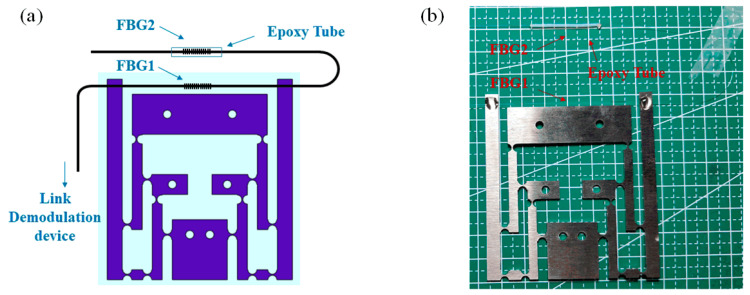
FBG temperature and strain simultaneous detection. (**a**) Schematic diagram; (**b**) concrete image.

The center wavelengths of the FBG strings were tested using a spectrum analyzer (AQ6374) and found to be 1544.7370 nm and 1549.7520 nm, respectively; their reflection spectra are shown in Figure 4. The design of the FBG temperature–strain detection structure is shown in Figure 5, where two FBGs with different wavelengths were etched on a string of FBGs with central wavelengths of approximately 1545 nm and 1550 nm. When FBG was stretched or the outside temperature rose, the reflected light center wavelength redshifted, and the opposite one blueshifted. An FBG with a central wavelength of approximately 1545 nm (FBG1) was placed on a hinged structure to measure the strain parameter. The output of the sensitized structure was uniformly attached with UV-curable adhesive (Norlan NO81), and the FBG was ensured to be in a tensile state when pasted. An FBG with a central wavelength of approximately 1550 nm (FBG2) was used for the temperature measurements. It was threaded into an epoxy tube slightly longer than the length of the grating area, with UV-curable adhesive on both sides, keeping the FBG area loose and not in contact with the tube wall. In such a design, the change in the center wavelength of FBG2 was only related to the change in the external temperature because it had been kept in a relaxed state and was not affected by the strain. Therefore, the temperature parameter on the surface of Li-ion batteries could be acquired by changing the center wavelength of FBG2. FBG1 and FBG2 were placed close to each other, and the temperatures of the two positions were considered to be the same, in a small range. According to Equation (9), when the temperature change around FBG1 was determined, its center wavelength change as a function of the temperature could be obtained. The total center wavelength change for FBG1 was then used to obtain the center wavelength change due to external strain, to establish the corresponding sensing relationship, and to realize strain demodulation.

### 2.3. Calibration of the FBG Sensor

**Figure 6:**
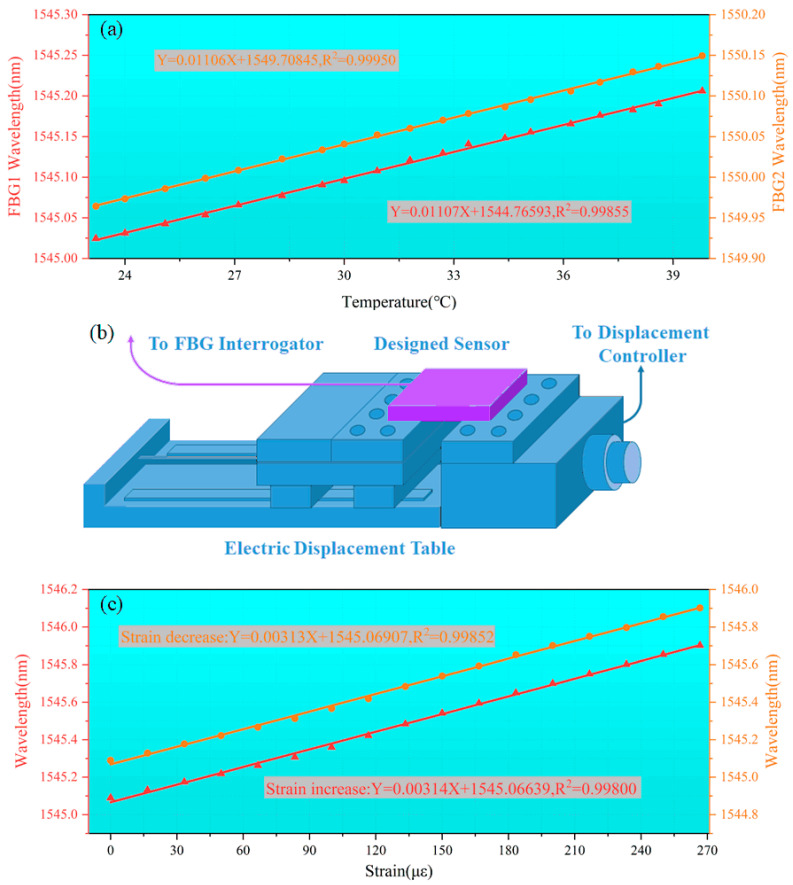
Calibration of (**a**) temperature- and (**c**) strain-sensing characteristics of the designed sensor; (**b**) strain calibration platform for sensors.

Calibration of the temperature detection characteristics of the designed sensor was carried out using a temperature–damp-heat test chamber (ATEC CH35C). The chamber temperature was set between 23 °C and 40 °C. An FBG demodulator (YOSC-FD-M, accuracy 0.2 pm, resolution 0.1 pm) was used to record the change in the wavelength t the center of each FBG after the temperature in the test chamber was stabilized at certain time intervals. The center wavelength change for FBG1 was used to measure the temperature interference characteristics of the strain-sensing grating, and the center wavelength change for FBG2 was used for the real-time sensing of the external temperature change because it was not subject to strain change. In the temperature calibration process, the calibration was based on the effect of the temperature around the FBG actually protected by the epoxy resin on its center wavelength. The center wavelength was directly and linearly related to the outside temperature of the FBG protected by the epoxy resin. Even if there was a possibility that the temperature measurement capability of the FBG after protection by the epoxy resin would be low, this was still reflected in the calibration process. Therefore, this calibration method would be meaningful for the acquisition of actual measured temperature data. As shown in Figure 6a, the temperature sensitivity of FBG1 appeared to be 11.07 pm/°C, and the temperature interference sensitivity of FBG2 appeared to be 11.06 pm/°C. Based on the resolution of the demodulator for conversion, the temperature resolution of FBG2 in the designed sensing system was found to be 0.01 °C, sufficient to meet the temperature detection requirements of Li-ion batteries. The two FBGs showed close sensitivities, and the sensor appeared able to effectively realize temperature detection in various operating states of the batteries.

A micron-level high-precision tensile displacement motorized stage (PTL-S100) was used to calibrate the strain detection characteristics of the designed sensor. The value of the strain change was obtained by calculating the amount of change in length produced by the displacement stage acting on the sensor relative to the change in the spacing of the original fixed points. The inputs of the sensitized structure were fixed on both sides of the displacement stage platform, guaranteeing a certain stretch, as shown in Figure 6b. The sensor and displacement stage were arranged in the temperature–damp heat test chamber to ensure that the surrounding temperature was room temperature (25 °C). The electric displacement stage was operated to record the center wavelength change for the FBG by shifting it by 1 μm at certain time intervals. Then, the electric displacement stage was shifted back 1 μm once, until the strain was zero (i.e., the platform was back to the original position). The central wavelength changes for the FBG at each step were determined and analyzed to obtain the strain detection characteristics, as shown in Figure 6c. It can be seen that the strain sensor showed good repeatability, with a strain sensitivity of 3.132 pm/με. Based on the resolution conversion of the demodulator, the strain resolution of FBG1 in the designed sensing system was 0.03 με, which is sufficient for the strain detection of Li-ion batteries. The strain sensitivity of the bare FBG was found to be 0.327 pm/με by arranging a displacement gripper (PT12) on the displacement stage table and stepping continuously in and out in the same manner and with the same initial fixation point spacing. The actual strain detection capability of the sensitized structure with the sensitization multiplier appeared to be increased by 9.578 times. These results were compared with the FEM simulation results and appeared to be basically the same. The reason that the results for the actual sensor in the presence of the sensitization multiplier were lesser than the simulation results might be the deformation of the glue used to fix the fiber to the output end of the sensitized structure. Comprehensively analyzed, The sensor can effectively realize the detection of surface strain under various operating conditions of the batteries.

### 2.4. Battery Testing Based on the Designed Sensor

Relevant battery tests were conducted using a PHEV NCM rechargeable Lithium-ion battery (FEPEC360030A), and the relevant parameters of the battery are shown in Table 2. The battery test system (BAT-NEEFLCT-05300-V010, FUJIAN NEBULA ELECTRONICS Co., Ltd., Fuzhou, China) was used to realize charge and discharge cycles under different conditions. A temperature–damp heat test chamber (ATEC CH35C) was used to ensure that the temperature of the battery cycle operation was maintained at 25 °C.

**Figure 7:**
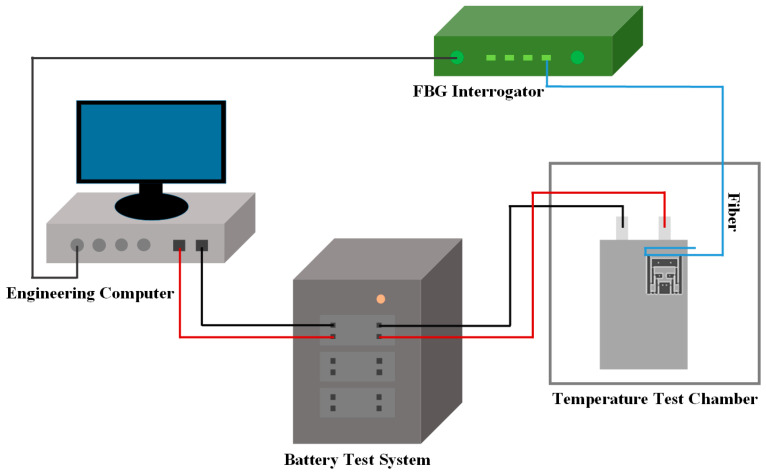
The constructed Li-ion battery test system based on the designed sensor.

The designed sensor was adhered to the cathode surface of the battery using Norlan NO61 type UV-curable adhesive. The FBG structure was glued to the output end of the hinge structure. The adhesion point was a small part of the structural piece at the input end of the designed sensitized structure, which was directly adhered to the plastic film on the surface of the battery. The entire lever and hinge structure of the sensitized structure, as well as the fixed plates and the dividers for mechanical support, were suspended to avoid the influence on the expansion of the battery itself. An FBG demodulator (YOSC-FD-M) was utilized to record the real-time center wavelength changes in each FBG in real time. The whole sensing and testing system is shown in Figure 7.

To study the temperature and strain detection performance of the designed sensor under normal operation of the Li-ion battery, charge and discharge tests of the Li-ion battery under regular operation conditions were conducted. In the first test, we performed a continuous CC Charge–CV Charge–CC Discharge–CC Charge cycle. First, a constant-current charge was performed at 30 A (1 C), with the voltage reaching 4.15 V. Then, a constant-voltage charge was performed at a constant voltage of 4.15 V by reaching a cutoff current of 1.5 A (0.05 C). At the end of the charging process, the Li-ion battery was discharged directly at a constant current of 30 A without resting until the voltage of the Li-ion battery was reduced to 2.8 V. The CC charging process was carried out immediately after the discharging process. The experiment was completed after several cycles. In the second test, we performed a continuous CC Charge–CV Charge–Rest–CC Discharge–Rest–CC Charge cycle, adding one hour of rest time between each charge and discharge.

To study how the surface temperature and strain changed during the operation of the Li-ion battery under different charging and discharging rates, a test of the Li-ion battery under different charging and discharging rates was carried out. This Li-ion battery had a maximum charging multiplier of 3 C and a maximum discharging multiplier of 5 C, and the charging and discharging strategy followed the CC Charge–CV Charge–Rest–CC Discharge–Rest cycle. That is, the constant-current charged up to the cutoff voltage, then the constant-voltage charged up to the cutoff current, with a resting time of two hours, and finally the constant-current discharged up to 2.8 V, with another two hours of resting. The first step was to perform a charge and discharge test up to 3 C, and the selected charge and discharge rates were 0.5 C, 1.0 C, 1.5 C, 2.0 C, 2.5 C, and 3 C. After that, multiple charge and discharge experiments were conducted with the charging rate maintained at 3 C and the discharging rate at 3.5 C, 4.0 C, 4.5 C, and 5 C.

## 3. Results and Discussion

### 3.1. Temperature and Strain Characterization of the Li-Ion Battery under Normal Conditions

**Figure 8:**
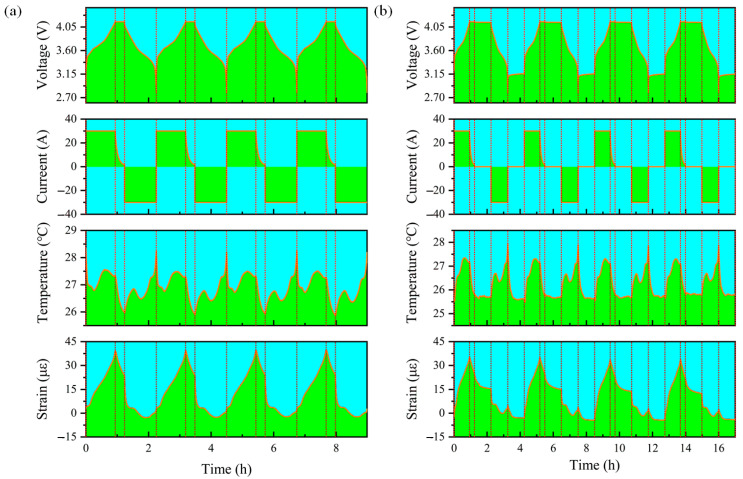
Characteristics of surface temperature and strain changes in the analyzed Li-ion battery (**a**) without and (**b**) with rest during conventional charge/discharge cycles.

During the CC Charge–CV Charge–CC Discharge–CC Charge process, four of the cycles were selected to analyze the temperature and strain changes, as shown in Figure 8a. Firstly, the trend of the temperature change was analyzed. The surface temperature of the Li-ion battery first decreased and then increased during CC charging. The drop here was because the battery temperature remained high. After all, there was no resting before the last constant-current discharge step of the previous cycle. The temperature drop at this point was not caused by the CC charge of the current cycle. Toward the end of the charge, however, the temperature at the surface of the Li-ion battery decreased slightly at this point, although the voltage increased more rapidly. During CV charge, the temperature on the surface of the Li-ion battery decreased rapidly, indicating that at this time, there was only a small heat generation or no heat generation. This could be due to the charging current gradually decreasing during constant-voltage charging, the internal molecular movement rate of the battery decreasing dramatically, the heat production decreasing, and the heat energy being released. During CC discharge, the temperature on the surface of the Li-ion battery rose dramatically. Although there was a period of temperature drop in the intermediate stage of the discharge, the temperature on the surface of the Li-ion battery rose dramatically in the late stage of the discharge when the voltage dropped dramatically, reaching the highest value of the whole continuous charge and discharge cycle. Secondly, the trend of the strain change was analyzed. The strain on the surface of the Li-ion battery showed an overall increase during CC charge, and there was a certain correlation between the trend of its change and the trend of voltage change. During CV charge, the strain on the surface of the Li-ion battery decreased, but at a diminishing rate, and the strain had a tendency to stabilize gradually. During CC discharge, the strain on the surface of the Li-ion battery decreased dramatically, then there was a stabilization period when the voltage decreased more slowly, and finally the strain decreased and then increased.

During the CC Charge–CV Charge–Rest–CC Discharge–Rest–CC Charge process, four of these cycles were also selected to analyze the temperature and strain changes, as shown in Figure 8b. Firstly, the trend of temperature change was analyzed. At the beginning of CC charge, the surface temperature of the Li-ion battery rose rapidly. Towards the end of charging, although the rate of voltage increase augmented, the temperature decreased slightly, which is consistent with the previous analysis during the continuous charging and discharging cycle. The surface temperature of the Li-ion battery decreased rapidly during CV charge and remained stable during the resting period. The trend of the temperature change during CC discharge was consistent with that during the previous cycle, and the peak temperature was lower than in the absence of resting, indicating that increasing the resting process had a positive effect on the safe and stable operation of the Li-ion battery. Secondly, the trend of strain change was analyzed. During CC charge, the trend of strain change was consistent with that observed during continuous charging and discharging. During CV charge, there was still a decrease in strain, and at this time, due to the increase in the resting period at the end of the charging, it was found that the strain tended to stabilize at a high level, which is consistent with the previous analysis. During DC discharge, the strain decreased dramatically, and there was a steady period when the voltage decreased more slowly. Then, the strain underwent a process of decreasing and increasing. During the resting process after cycling, the surface strain of the battery continued to decrease as the temperature returned to room temperature, and the lowest value of the strain was lower than that measured in the previous discharge period. Therefore, it was proved that the increase in the strain at the end of the discharge period was related to the thermal expansion produced by exothermic heat.

### 3.2. Temperature and Strain Characterization for the Li-Ion Battery under Different C-Rates

**Figure 9:**
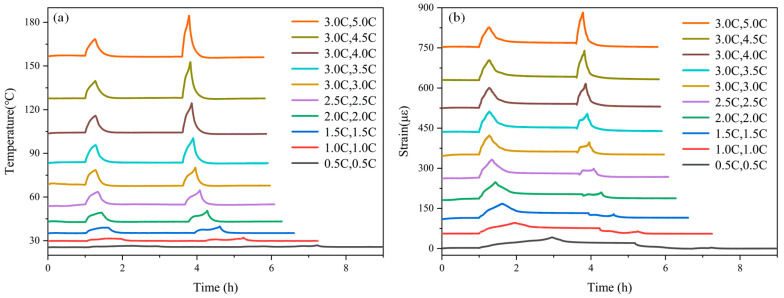
Offset stacked line plots of surface (**a**) temperature and (**b**) strain variation of the Li-ion battery at different charging and discharging rates.

**Figure 10:**
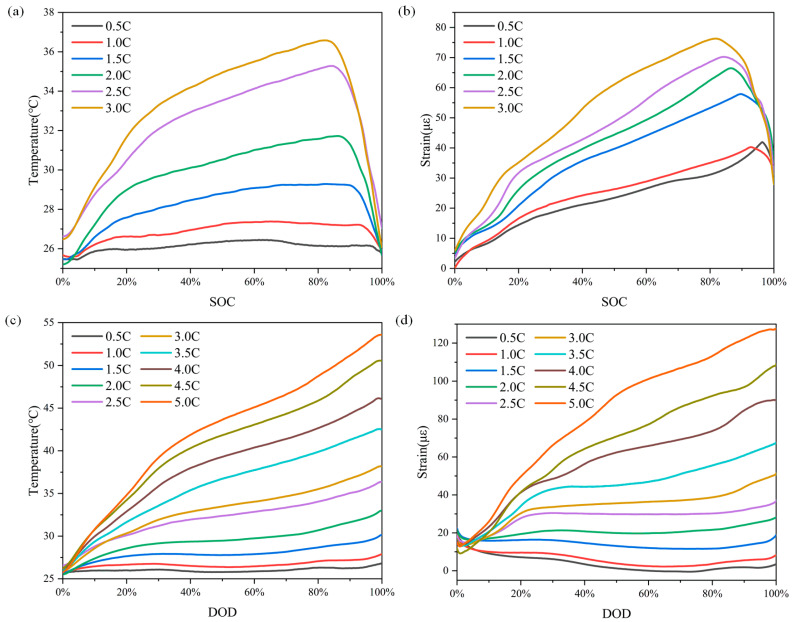
The curves of (**a**) temperature and (**b**) strain relative to SOC changes at different charging and discharging rates. The curves of (**c**) temperature and (**d**) strain relative to DOD changes at different charging and discharging rates.

The temperature and strain variation curves of the Li-ion battery in the CC Charge–CV Charge–Rest–CC Discharge–Rest–CC Charge cycle were analyzed for different charging and discharging rates from 0.5 C to 5 C. The offset stacked line plots are shown in Figure 9. During the charging process, the temperature and strain on the surface of the Li-ion battery increased dramatically with the increase in the charging rate. At the end of charging at different rates, the strain on the surface of the Li-ion battery reached a stable value, which was almost the same at different charging and discharging rates. Combined with the temperature changes on the surface of the Li-ion battery at different rates, the thermal expansion caused by the temperature rise in the charging process was the main factor that affected the peak strain on the surface of the Li-ion battery. During the discharge process, with the increase in discharge rate, the temperature and strain on the surface of the Li-ion battery increased dramatically. The temperature peak was larger than that obtained when charging at a high rate. At the end of the discharge, the strain decreased to the initial value before charging. The increase in strain during the discharge process depended on the thermal expansion caused by the increase in the temperature. The curves of temperature and strain relative to SOC and DOD during charging and discharging are plotted in Figure 10. During charging, the temperature and strain of the Li-ion battery increased with the increase in SOC, and both temperature and strain decreased at the end of charging. Decreasing temperature and strain were observed during CV charge. Both temperature and strain during discharge increased with the increase in DOD, and the decreasing trend of strain was almost covered by thermal expansion during high-rate charging and discharging, due to the more drastic temperature change.

## 4. Conclusions

In summary, a sensor based on a hinged structure, using differential lever sensitization and optical fiber sensing for simultaneous temperature and strain measurement was designed. After calibration, the temperature- and strain-sensing performance of the sensor was verified. The sensor also realized real-time contact sensing of the surface temperature and strain change characteristics of a Li-ion soft-pack battery. During the regular charge and discharge cycles of a Li-ion battery, both charge and discharge were exothermic processes. However, the overall changes in the battery temperature were not significant, and the temperature peak occurred at the end of a constant-current discharge. The strain changes on the surface of the Li-ion battery were related to the electrochemical changes during the charging and discharging of the battery itself and the thermal expansion or contraction caused by the temperature changes during charging and discharging. The charging process showed an increasing strain, and the discharging process showed a decreasing strain. During the charging and discharging of the Li-ion battery at different rates, the battery surface temperature changes began to be dramatic. The higher the charge and discharge rate, the higher the peak surface temperature of the Li-ion battery. The heat release during discharge was larger than that during charge. The surface strain changes of the Li-ion battery at a high charge and discharge rate mainly depended on the thermal expansion. The strain changes caused by electrochemical changes at different charge or discharge rates were basically the same. In contrast to the conventional optical fiber sensing-based surface temperature and strain measurement scheme for Li-ion batteries, this present study provides a higher sensitivity scheme for the simultaneous measurement of the two parameters, taking into account thermal expansion and electrochemical changes as strain-inducing factors.

## Figures and Tables

**Table 1:** Summary table of the sensing status of some FBG used in Lithium-ion soft-packed batteries.

Major Authors	Sensitivity	Measurement Range	Resolution
Peng et al. [30]	11.55 pm/με	0–300 με	1 pm (interrogator)
Susana et al. [32]	~8.55 pm/°C	0–35 °C	0.12 pm (interrogator)
Nascimento et al. [33,34]	7.43 pm/°C and 0.83 pm/µε	5–50 °C	5 pm (interrogator)
Sommer et al. [35,36,37]	10 pm/°C and 1 pm/µε	/	0.1 °C, 1 µε
Ee et al. [31]	1 pm/µε and 11 pm/°C	The wavelength shift of 80–104 pm	/

**Table 2:** Parameters of the PHEV NCM rechargeable Lithium-ion battery.

Item	Specification
Nominal capacity	30 Ah (1.0 C discharge at 25 °C, 4.15–2.80 V)
Operation voltage range	2.80–4.15 V
Standard charge current	Standard: 30 A (1.0 C)
Maximum continuous charge current	90 A (3.0 C)
Maximum continuous discharge current	150 A (5.0 C)
Cell dimension	Length × width × thickness: 232.0 × 164.0 × 7.48 mm (30% SOC, 90 kgf Flat pressure test)
Operating temperature	Charge: 0–55 °C, discharge: −30–55 °C

## Data Availability

Data are available on request from the authors.
